# Evaluation of Zika rapid tests as aids for clinical diagnosis and epidemic preparedness

**DOI:** 10.1016/j.eclinm.2022.101478

**Published:** 2022-06-04

**Authors:** Debi Boeras, Cheikh Tidiane Diagne, Jose L. Pelegrino, Marc Grandadam, Veasna Duong, Philippe Dussart, Paul Brey, Didye Ruiz, Marisa Adati, Annelies Wilder-Smith, Andrew K. Falconar, Claudia M. Romero, Maria Guzman, Nagwa Hasanin, Amadou Sall, Rosanna W. Peeling

**Affiliations:** a Global Health Impact Group, Atlanta, USA; b Institut Pasteur de Dakar, Dakar, Senegal; c Instituto Pedro Kouri, Havana, Cuba; d Institute Pasteur du Laos, Vientiane Laos; e Institute Pasteur du Cambodge, Phnom Penh, Cambodia; f Institut Pasteur de Madagascar, Antananarivo, Madagascar; g National Institute for Quality Control in Health, Rio de Janeiro, Brazil; h Umea University, Umea, Sweden; i Clinical Research Department, London School of Hygiene and Tropical Medicine, Keppel Street, London WC1E 7HT, UK; j Universidad del Norte, Barranquilla, Colombia; k Supply Division, UNICEF, Copenhagen, Denmark

**Keywords:** Zika, Diagnostics, Clinical medicine, Epidemic preparedness, Evaluation, Advance purchase commitment, Biobanking network

## Abstract

**Background:**

Development and evaluation of diagnostics for diseases of epidemic potential are often funded during epidemics, but not afterwards, leaving countries unprepared for the next epidemic. United Nations Children's Emergency Fund (UNICEF) partnered with the United States Agency for International Development (USAID) to address this important gap by investing in an advance purchase commitment (APC) mechanism to accelerate the development and evaluation of Zika rapid diagnostic tests (RDTs) for case detection and surveillance. This paper describes the performance evaluation of five Zika RDTs eligible for procurement.

**Methods:**

A network of European Union-funded ZikaPLAN sites in Africa, Asia, Latin America with access to relevant serum specimens were selected to evaluate RDTs developed for the UNICEF APC mechanism. A standardised protocol and evaluation panels were developed and a call for specimens for the evaluation panels issued to different sites. Each site contributed specimens to the evaluation from their biobank. Data were collated, analysed and presented to the UNICEF Procurement Review Group for review.

**Findings:**

Three RDTs met the criteria for UNICEF procurement of sensitivity and specificity of 85% against a refence standard. The sensitivity/specificity of the ChemBio anti-Zika Virus (ZIKV) immunoglobulin M (IgM) test was 86.4 %/86.7% and the ChemBio ZCD system for anti-ZIKV IgM was 79.0%/97.1%, anti-dengue virus (DENV) IgM 90.0%/89.2%, anti-Chikungunya virus (CHIKV) IgM 90.6%/97.2%. The sensitivity/specificity of the SD Biosensor anti-ZIKV IgM was 96.8 %/90.8%, anti-DENV IgM 71.8%/83.5%, the DENV nonstructural protein 1 (NS1) glycoprotein 90.0%/90.2%, anti- yellow fever virus (YFV) IgM 84.6%/92.4%, anti-CHIKV IgM 86.3%/97.5%.

**Interpretation:**

Three RDTs fulfilled the performance thresholds set by WHO and were eligible for UNICEF procurement. These tests will improve the diagnosis of ZIKV and other arboviral infections as well as providing countries with better tools for surveillance and response to future epidemics.

**Funding:**

This work was supported by the USAID grant GHA-G-00-07-00007 and ZikaPLAN (European Union's Horizon 2020 Research and Innovation Programme under Grant Agreement No. 734584).

**Research in context**

**Evidence before the study**

We searched MEDLINE and PubMed on July 31 2021 and references from relevant articles on September 30, 2021 for the performance of IgM rapid diagnostic tests (RDTs) for Zika virus infections using the search terms “Zika”, “IgM”, “serologic”, “tests”, “evaluation” and “diagnostics” for articles published in English between Jan 1, 2015 and September 30, 2021. We found 56 articles, of which only two articles reported independent evaluation of the performance of two Zika immunoglobulin M (IgM) rapid tests, none of which were found to have satisfactory performance.

**Added value of this study**

This work presents data on the independent evaluation of three Zika RDTs made by two companies under the United Nations Children's Emergency Fund (UNICEF) Advanced Purchase Commitment (APC) funding mechanism to incentivise companies to continue the development of tests after the Zika epidemic was over. The evaluations were performed using well-characterised specimens archived at a pre-established network of quality laboratories in low- and middle-income countries as part of the EU-funded ZikaPLAN biobanking and evaluation network. One anti-Zika IgM test and two multiplex tests that can detect IgM antibodies against Zika and other arboviral infections were found by the UNICEF Procurement Review Group to have satisfactory performance and eligible for UNICEF procurement for use in countries as surveillance and case detection tools.

**Implications of all the available evidence**

This study suggests the value of the APC mechanism to incentivise companies to continue the development of tests needed for epidemic preparedness beyond the current epidemic. A pre-established quality-assured biobanking and evaluation network of laboratories in Zika virus endemic countries expedited the independent evaluations of diagnostics to ensure that they are fit for purpose as case detection and surveillance tools. This model has now been adopted by the Africa Centres for Disease Control and Prevention as part of their epidemic preparedness plan for the continent.

Alt-text: Unlabelled box

## Introduction

Development and evaluation of diagnostics for diseases of epidemic potential are often funded during epidemics and left unfinished once the epidemic is over, leaving countries ill-prepared to combat the next epidemic.^1^^,^^2^

Accurate diagnostic tests for arboviral infections play a critical role in case detection and surveillance to provide early warning of potential outbreaks.^3^ The recent introduction of Chikungunya virus (CHIKV) and Zika virus (ZIKV) into dengue virus (DENV) endemic areas has created new challenges for clinical management and public health response worldwide. These three arboviral diseases has similar clinical presentations during the acute phase of illness. The use of molecular tests to detect DENV and ZIKV genes is limited by the low viral load in blood and a very transient period of viremia. Serological diagnosis is complicated by extensive cross-reactivity between anti-DENV and ZIKV immunoglobulin M/ immunoglobulin G (IgM/IgG) antibodies.^4^, ^5^, ^6^, ^7^ Antibodies produced in primary infection tend to have higher specificity compared to those produced in secondary infections, further reducing the utility of serology tests for the diagnosis of flavivirus infections in areas where these infections are endemic.^4^, ^5^, ^6^, ^7^ A systematic review showed that about 15–84% of antibodies produced against non-DENV flaviviruses were cross-reactive with those of DENV on different assays.^8^ As a result, the World Health Organization recommended that an anti-ZIKV IgM positive test results should be interpreted as recent infection with a member of the flavivirus family unless the result is confirmed by a Plaque Reduction Neutralization Test (PRNT), which, unfortunately, is not widely available to support clinical case management.^9^

The association of ZIKV infections with congenital birth defects, including microcephaly, and other neurologic complications and its potential spread through sexual transmission has made the diagnosis and surveillance of ZIKV infections an important priority, especially for case management and counselling for pregnant women^10^, ^11^, ^12^ As soon as ZIKV was declared a public health emergency of international concern (PHEIC), more than 50 companies and many more academic institutions raced towards developing more sensitive and specific diagnostic tests.^1^

After WHO declared that ZIKV was no longer a PHEIC, new laboratory-based diagnostic assays were reported in the published literature but accurate, affordable and accessible diagnostic tests that can be used by health providers in community settings were not available.^13^, ^14^, ^15^, ^16^, ^17^, ^18^ The Office of Innovation at the United Nations Children's Emergency Fund (UNICEF) conducted a landscape of diagnostic tests for Zika and decided to collaborate with the United States Agency for International Development (USAID) on an initiative to incentivize the development, evaluation and scale up of rapid, low-cost, sensitive and specific anti-ZIKV IgM rapid diagnostic tests (RDTs), as well as multiplex IgM RDTs that could be used to distinguish between ZIKV infections with those caused by other arboviruses, such as DENV, yellow fever virus (YFV) and CHIKV to ensure that effective and affordable tests for ZIKV can become commercially available for use at community level in the fastest possible timeframe.^19^ These tests should be simple to use at the point-of-care (POC) and yield results within 10–30 min. UNICEF and USAID developed an Advance Purchase Commitment (APC), which aims to reduce demand uncertainty risks for manufacturers who invest in research and development towards new products. Solicitations for the evaluation of products as part of the APC initiative was conducted across two rounds of calls from 2017 to 2019. Following the completion of two independent tender rounds, UNICEF signed six conditional Long-Term Agreements (LTAs) for the procurement of ZIKV singleplex (ZIKV test only) and multiplex (ZIKV and other arboviruses) RDTs, including access to an APC for procurement during 2019–2020.

Under these agreements, procurement was conditional on satisfactory performance of the new products in independent evaluations relative to standards set out by WHO and UNICEF^20^ During the ZIKV PHEIC, the London School of Hygiene and Tropical Medicine (LSHTM) received funding from the European Horizon 2020 programme to set up a network of biobanking sites in different geographical regions and to coordinate the collection of well-characterized specimens that can be used to accelerate the development and evaluation of ZIKV tests to aid in diagnosis and surveillance.^21^^,^^22^ This network of biobanking sites provided samples for the independent evaluation of ZIKV tests that were eligible for APC procurement.^23^ LSHTM coordinated the evaluation using the same system developed by the UNICEF/UNDP/World Bank/WHO Special Programme for Research and Training in Tropical Diseases (TDR) for the evaluation of DENV IgM and nonstructural protein 1 (NS1) tests.^24^^,^^25^

This paper describes the results and significance of these evaluations as an important component of an innovative financing mechanism for test development to ensure that countries have diagnostic tools for clinical management of ZIKV infections and for surveillance, providing early alert against future outbreaks.

## Methods

### Tests evaluated

A total of five different anti-ZIKV IgM RDTs from three companies were submitted for evaluation after two tender rounds. Some of the tests submitted in the second round were slightly modified versions of tests assessed in the first round. Tests were either singleplex or multiplex – a singleplex test refers one that detect a single test target only, which in this case is ZIKV, while a multiplex test refers to one that can detect several different targets, which in this case are ZIKV plus other arboviruses transmitted by the same Aedes mosquito species. For this tender, UNICEF adopted the World Health Organization (WHO) Target Product Profile (TPP) for ZIKV diagnostics and set mandatory requirements of 85% for sensitivity and specificity of ZIKV singleplex and multiplex tests compared to a laboratory reference standard.^20^ Results of tests that were not yet commercialized are not reported here as companies were using the results of the evaluation to improve their test. We report the results of three RDTs from two companies that were selected for procurement by the UNICEF Procurement Review Group for use in low- and middle-income countries.

The focus of this evaluation is on immunoglobulin M (IgM) tests which can be used by countries as an aide for case detection, surveillance and outbreak alert. In a ZIKV-infected patient, anti-ZIKV IgM antibodies can usually be detected within the first 2 weeks of symptoms and continue to be detectable in some patients for as long as 5 or 6 months.^4^^,^^11^^,^^12^ The presence of anti-ZIKV IgM antibodies can be interpreted as suggestive of a recent infection but not of an acute infection because of the persistence of these antibodies. An outbreak is suspected when more people test positive for IgM or there are higher than normal levels of IgM in a population. Immunoglobulin G (IgG) antibodies provide a marker of exposure and can be used to estimate the extent and geographic distribution of an outbreak. Two of the RDTs can be used to detect IgG antibodies but, as the focus of this evaluation is on the use of anti-ZIKV IgM antibody tests, the evaluation of the IgG components of these tests will not be reported in this study.

**Figure 1:**
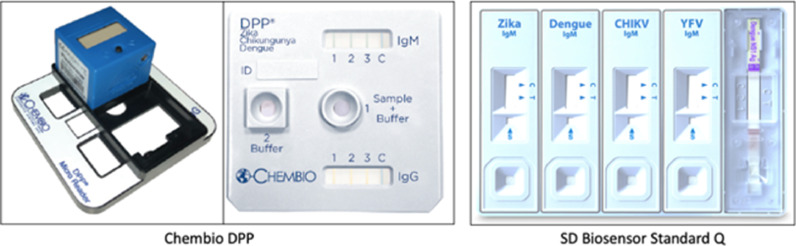
**Chembio Diagnostics DPP® ZCD IgM/IgG (Zika/Chikungunya/Dengue IgM/IgG) System and SD Biosensor (SDB) STANDARD Q Arbo Panel Test.** The Chembio ZIKV, CHIKV, and DENV multiplex serology assay system consists of a single cassette where the sample and buffer are placed are placed into a well marked 1 and a second buffer added into well 2. The results are read by placing a RFID microreader over the IgM and IgG windows where result for Zika antibodies can be read in Line 1, chikungunya virus antibodies in Line 2, dengue virus antibodies in Line 3 and C is the control line. For the Chembio Diagnostics DPP® Zika IgM/IgG System, the test cassette is similar to the ZCD IgM/IgG system except that each result window only contains 2 lines, one for the zika antibodies and the other for the control line (picture not shown) . SD Biosensor (SDB) STANDARD Q Arbo Panel Test: the SD Biosensor assay is an immunochromatographic assay for the detection of Dengue NS1 Antigen and IgM antibodies to Zika/Dengue/Chikungunya/Yellow fever virus. It consists of 5 separate cartridges, where each requires specimen and buffer addition. The results can be read visually.

The Chembio Dual Path Platform (DPP)® Zika IgM/IgG System is a rapid 10–15 min immunochromatographic test for the detection and differentiation of IgM and IgG antibodies to ZIKV in 10 µl fingerstick whole blood, Ethylenediaminetetraacetic acid (EDTA) anticoagulated venous whole blood, serum, or EDTA-anticoagulated plasma samples (Figure 1). Results were read 10–15 min after adding buffer. The test is only valid if the control line (C) is present. The top test strip (window labeled T1) is for the detection of IgM antibodies to ZIKV and the bottom test strip (window labeled T2) is for the detection of IgG antibodies to ZIKV. The result scoring for IgM (T1) and IgG (T2) ZIKV-specific antibodies is performed with a specific Chembio developed Radio Frequency Identifier Driven (RFID) micro-reader. The configuration file on the RFID card carries the parameters specific to the detection of the test. The reader verifies the presence of the control line and measures color intensity followed by the interpretation of that test line as Reactive (“R”), Non-reactive (“NR”), the absence of a control line is interpreted as Indeterminate (“IND”) or Invalid (“INV”). The absence of a control line indicates that the specimen has not migrated down the nitrocellulose strip past the test line or the control line. The test is therefore considered IND or INV.

The Chembio DPP® ZCD IgM/IgG (Zika/Chikungunya/Dengue IgM/IgG) System is a similar rapid immunochromatographic test for the detection and differentiation of IgM and IgG antibodies to ZIKV; the detection and differentiation of IgM and IgG antibodies to CHIKV; and the detection and differentiation of IgM and IgG antibodies to DENV (Figure 1).

The top and bottom window lines labeled 1, 2, 3 are for the detection of IgM or IgG antibodies against DENV, ZIKV and CHIKV, respectively while line C is the control line. The reading of the results is similar to the Chembio DPP® Zika IgM/IgG System. Results are read 10–15 min after adding buffer and using the DPP micro reader and cassette adapter, as the order of the test lines in the readout windows may be different than the order of the letters Z, C and D in the product name. The DPP ZCD Micro Reader has been individually adapted for specific use with the DPP ZCD IgM/IgG Assay System.

The SD Biosensor (SDB) STANDARD Q Arbo I (Z/D/C/Y) Panel Test is an immunochromatographic lateral flow test to detect anti-ZIKV, CHIKV, DENV or YFV IgM specific antibodies and the DENV nonstructural-1 (NS1) glycoprotein in human whole blood, serum and plasma. The test is comprised of five separate cartridges (Figure 1). Each cartridge requires 10 uL of sample (whole blood, serum or plasma), followed by the addition of 3 drops of assay diluent buffer. Results are read in 15 min but cannot be read after 20 min. For the evaluation, all tests were performed in accordance with manufacturer's instructions. A test result is only valid if the control line is present.

All three assays described are intended for use in clinical and POC settings to aid in the differential diagnosis of infection in patients with clinical symptoms consistent with ZIKV infection. A recent history of travel to geographic regions during a period of active ZIKV transmission at the time of travel; and/or other epidemiologic criteria for which ZIKV testing may be indicated as part of public health response. These tests are intended to provide a preliminary result. Results of these tests should therefore not be used as the sole basis of patient management decisions and should be used in combination with clinical observations, patient history, epidemiological information, and other laboratory determined criteria. The results must be confirmed by using the current CDC or local guidelines for the diagnosis of ZIKV or other arboviral infections.

### Selection of evaluation sites

LSHTM has developed a network of specimen and strain biobanks and diagnostic evaluation sites as part of the European Union funded project, ZikaPLAN.^21^^,^^22^ The goal of this ZikaPLAN diagnostic work package has been to accelerate the development and evaluation of ZIKV diagnostics for clinical and surveillance use through the establishment of a network with access to well-characterized clinical specimens and capacity for conducting independent evaluations.

To qualify, all sites must be proficient in performing reference standard testing for ZIKV and other arboviruses and compliant with Good Clinical Practice/Good Clinical Laboratory Practice.^23^ Besides being quality-assured, all sites agreed to a set of guiding principles that ensures equitable access, transparency of all procedures, respect for ethics and national laws, confidentiality of those who contribute samples and fair sharing of benefits.^23^ Sites should have biobanks with well-characterised serum samples that can be used for evaluations and have ethical approval for the use of specimens for diagnostic research, including test evaluations. All sites within the network agreed to use a standardised protocol for the evaluation of ZIKV diagnostics against reference standard assays.

At the time of the call for specimens for the UNICEF evaluation, three sites from the ZikaPLAN network responded: the Institut Pasteur Dakar, Senegal (IPD), Instituto Pedro Kouri, Habana, Cuba (IPK), and the Universidad del Norte, Barranquilla, Colombia (UNO). IPD and IPK are both WHO Collaborating Centres with biobanks of well-characterised patients’ ZIKV and DENV positive and negative specimens and specimens from individuals vaccinated against the Yellow Fever Virus (YFV). IPD have specimens from surveillance and outbreak investigations of infections caused by haemorrhagic fever viruses. IPK and UNO have large numbers of well-characterised anti-DENV specimens of all serotypes.

Additional sources of well-characterised specimens for CHIKV IgM positive specimens had to be sought as most of the biobanking and evaluation sites within the ZikaPLAN network did not have access to sufficient numbers of confirmed specimens to perform the evaluations of the CHIKV components. Two additional sites with CHIKV-positive samples were therefore identified – Institut Pasteur in Vientiane, Laos (IPL) and Institut Pasteur du Cambodge in Phnom Penh, Cambodia (IPC). Site assessment visits to the IPC and IPL were conducted in April 2019 and both sites fulfilled the quality criteria to join the biobanking and evaluation network.

In Brazil, the Chembio DPP ZCD assay had already received Agência Nacional de Vigilância Sanitária (ANVISA) regulatory approval. This data was shared by the FioCruz National Institute of Quality Control (INCQS) and INCQS was included as part of the ZikaPLAN network. The origins of the samples used for this evaluation at each site are summarised in Table 1.

**Table 1:** Origins of specimens used in the evaluation panels.

	Anti-ZIKV	Anti-DENV	Anti-YF	Anti-CHIKV
**Institut Pasteur Dakar (IPD), Senegal**	IgM and IgG: Cape Verde Islands; IgG: Guinée Bissau	IgM: Touba, Senegal 2018; IgG: Tambacounda, Senegal 2013	IgM: 2017–8 Nigeria outbreak IgG: Kédougou, 2012 Senegal vaccination coverage study	IgM: Kédougou, Senegal, 2015 IgG: Soudan, 2012
**Institut Pasteur Laos, (IPL) Vientiane, Laos**				IgM and IgG positive samples collected for national arbovirus surveillance, Laos
**Institut du Cambodge (IPC), Phnom Penh, Cambodia**		IgM: surveillance samples, from across Cambodia		IgM: surveillance samples, from across Cambodia
**Instituto Pedro Kouri, (IPK), Havana, Cuba**	Samples collected for national arbovirus surveillance		Samples from vaccinated individuals	
**Universidad del Norte (UNO) Barranquilla, Colombia**	Samples collected for pathogenesis studies			
**Instituto Nacional de Controle de Qualidade em Saude (INCQS) Rio de Janeiro, Brazil**	Samples from surveillance and research studies at the Fundacao Oswaldo Cruz (Fio Cruz) branches across Brazil.			

ZIKV: zika virus; DENV: dengue virus; CHIKV: chikungunya virus; YF: Yellow Fever virus.

### Design of the evaluation

**Figure 2:**
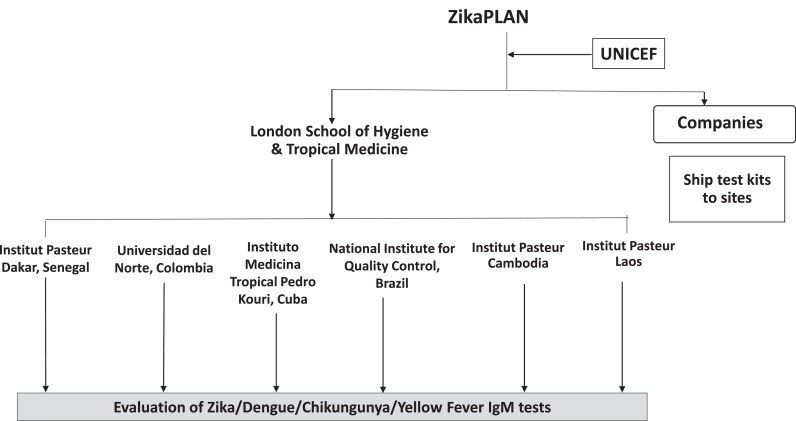
**ZikaPLAN network of biobank and evaluation sites.** The evaluation of Zika virus Rapid Diagnostic Tests (RDTs) submitted under the UNICEF tenders took place across a global network to source needed specimens. Text box on left shows steps in evaluation process. The London School of Hygiene & Tropical Medicine (LSHTM) provided the overall coordination. Triage using the ´Sudden Death´ panel was only performed at IPD.

When a test was submitted for evaluation, evaluation panels were designed by LSHTM according to the intended use, diagnostic target and the format of the test. A call for specimens went out to all the sites to identify sites having specimens that they were willing to contribute towards the evaluation panel (Figure 2). Each site had a unique set of archived specimens available to contribute for test evaluations. Tests were sent by the companies to each site for the evaluation which avoided the difficulty and high cost of sending infectious materials across borders. Multiple sites contribute data towards the full evaluation.

All tests under evaluation were assessed at two or more sites to ensure adequate geographic representation of the specimens in the evaluation panel and also to ensure the evaluation of the operational characteristics, such as ease of use were assessed by at least two teams of test users.

The sites were reimbursed by the companies for all the cost associated with the evaluations. Each site entered into an agreement with the companies and invoiced them for the expense of performing the evaluation on a cost recovery basis plus an estimated cost of replenishing the specimens used from its biobank.

### Defining the evaluation panels

The design of our ZIKV evaluation panel was based on that of the dengue evaluation panel recommended by the TDR Expert Advisory Committee on performance evaluations of arboviruses.^24^^,^^25^ The sensitivity panel for the first evaluation of ZIKV IgM tests at the IPD site consisted of 44 sera (20 high, 13 medium and 11 low concentrations of anti-ZIKV IgM antibodies, as characterised by the MAC-ELISA developed by IPK and the IPD based on the CDC MAC-ELISA with Cut-offs for Low as <6; Medium: > 6 to < 20; High: > 20. The inclusion of more sites allowed the network to use larger evaluation panels but maintaining the ratio of 60% specimens with low and medium titres to 40% of specimens with high titres to allow better discriminations between the comparative sensitivities of the tests under evaluation. Similar panels were designed for the evaluation of sensitivity of anti-DENV, anti-YFV and anti-CHIKV IgM tests. The analytical sensitivity of the SD Biosensor NS1 assay was evaluated by performing serial dilutions of commercial preparations of DENV NS1 from all four serotypes and determining the limit of detection of the SD Biosensor NS1 assay against these preparations.

### Specificity or challenge panel

The specificity of the RDTs under evaluation was assessed using challenge panels composed of sera from patients with infections that may cause potential cross-reactive antibody responses or conditions that could produce interfering substances such as rheumatoid factor autoantibodies produced from patients with rheumatoid arthritis. Table 2 shows the minimum number of specimens recommended by the technical advisory panel for these challenge panels. Finding sufficient number of IgM positive sera against YFV may be problematic. The specificity of the SD Biosensor STANDARD Q Dengue NS1 test was determined in a series of specificity panels: NS1 positive samples of YFV, ZIKV, West Nile Virus (WNV) and against fever syndromic similar samples from CHIKV and malaria, followed by dilutions of YFV NS1 and ZIKV NS1, and in a series of samples spiked with dilutions of flavivirus NS1 of ZIKV, of YFV, of WNV, and finally against spiked samples of CHIKV and malaria.

**Table 2:** Minimum number of specimens for anti-ZIKV, anti-DENV and anti-CHIKV IgM antibody specificity panels.

	Anti-ZIKV IgM	Anti-DENV IgM	Anti-CHIKV IgM
**Serum description**	n=		
Anti-ZIKV/DENV IgG POS	10	10	NA
**Heterologous flavivirus illnesses**			
Anti-ZIKV POS	-	30	30
Anti-DENV IgM POS	27	-	30
Anti-YFV IgM POS	15	15	15
**Other arbovirus illnesses**			
Anti-CHIKV IgM POS	10	10	NA
**Healthy donor samples**			
NEG to all*	10	10	10
***Subtotal***	***80***	***80***	***85***
**Febrile illnesses**-			
Malaria tick drop POS	19	19	19
**Systemic conditions**			
Rheumatoid Factor	17	17	17
***Subtotal***	***36***	***36***	***36***
**TOTAL**	**116**	**116**	**121**

NEG to all*: samples were negative in IgM and IgG assays to YFV, DENV, WNV, CHIKV and ZIKV as well as Rift Valley fever virus and Crimean Congo haemorrhagic fever virus. all anti-DENV IgM and anti-YFV IgM positive samples were tested by other flavivirus IgM ELISA (ZIKV, DENV and YFV) to rule out possible other flavivirus infection.

ZIKV: zika virus; DENV: dengue virus; CHIKV: chikungunya virus; YFV: Yellow Fever virus; WNV: West Nile virus.

### Evaluation process

Since well-characterised patients’ serum samples are a precious resource and are often only available in small volumes, the consensus by the network was that the evaluation should be divided into two stages so that these precious samples may be conserved to maximize the number of evaluations which could be performed and streamline a more expedited evaluation process. A test under evaluation was first assessed at IPD against small panels of healthy controls which were negative for all arboviruses. If a test passed this Sudden Death Panel with a score of 80% for specificity, then the laboratories would proceed with the full anti-ZIKV IgM evaluations. If the test scored less than 80% with this Panel, then the evaluation would not proceed further, hence the term, ‘Sudden Death Panel´.

### Sudden death panel

The Sudden Death Panel for the anti-ZIKA IgM antibody test comprised of 10 serum samples from healthy donors and 10 anti-ZIKV IgG positive/IgM negative samples. The anti-ZIKV IgG positive samples were confirmed by obtaining ZIKV-positive, but DENV, YFV and West Nile virus (WNV) negative 90% plaque reduction neutralization test (PRNT) at 1/8 specimen dilutions.^26^, ^27^, ^28^, ^29^, ^30^

The healthy donors’ samples were serum samples characterized as negative for both IgM and IgG antibodies against the following seven viruses: ZIKV, DENV, CHIKV, WNV, YFV, Rift Valley fever virus (RVFV) and Crimean Congo haemorrhagic fever virus (CCHFV) using IgM-capture ELISAs (MAC-ELISAs).^27^^,^^28^ The performances of the Chembio DPP® Zika IgM test, the Chembio DPP® ZCD IgM test, and the SD Biosensor STANDARD Q Arbo Panel test were each evaluated at four biobanking/evaluation laboratory sites across Africa, Latin America and Asia using a total of 1,060 well-characterised specimens to develop Sudden Death, sensitivity and specificity panels to evaluate the products. All products reported here passed Sudden Death (score > 80%) and proceeded to full evaluations. Sudden Death results were included in full evaluations.

### Reference tests

IgM reference methods used by IPD for ZIKV and other arboviruses were assays used at WHO Collaborating Centres which included the US Centers for Disease Control and Prevention (CDC) MAC ELISA, with specificity confirmed by PRNT.^29^ The reference assays for DENV used at IPK and IPC were standardized against the CDC MAC ELISA as these centres were part of the TDR Dengue Test Evaluation Network.^24^^,^^25^ The CHIKV reference assay at the IPC site was the assay used for the Laos national arbovirus surveillance.^31^ The INCQS in Brazil is the National Institute for Quality Control and has also standardized their IgM assays against the CDC MAC ELISA. The reference assays used to characterize specimens for dengue virus research at UNO site are as previously described.^32^^,^^33^

### Collation and analysis of results

The LSHTM was responsible for the collation of results across the ZikaPLAN network. All statistical analyses are performed in collaboration with IPD using R.4.1.0 software (2021-05-18). The  Statistical method used to quantify uncertainty is the Binomial Test. It's an exact test of a null hypothesis about the probability of success in a Bernoulli experiment. We use the R-fonction binom.test()  to perform this test.  This fonction provides  several values ​​including the probability of success under the null (0.5), alternative hypothesis (two.sided) and the confidence intervals for the probability of success. Confidence intervals are obtained by a procedure first given in Clopper and Pearson (1934).

The specificity of these RDTs were analysed against other arboviruses. Values for overall specificity included test specificity against arboviruses as well as against healthy controls and IgG positive specimens where available. The specificity values obtained in this evaluation were intended to alert users of these serologic test of potential cross-reactivity against arboviruses and were not meant to be interpreted as representing specificity of these tests in any real-life settings. Similarly the evaluation of these RDTs against specimens positive for malaria and rheumatoid arthritis are intended to inform manufacturers of non-specific binding events which they need to mitigate. The results were shared with manufacturers and they were given 30 days to respond with comments or questions. In collaboration with the ZikaPLAN Scientific Steering Committee, data was analyzed for presentation to UNICEF and USAID, as well as other UNICEF designated stakeholders.

### Ethics

This study is a laboratory-based evaluation which used left over samples from surveillance, outbreaks and research from the network of biobanking and evaluation sites. The laboratories in the network have institutional approval for the use of left-over samples for diagnostics evaluations. All the samples were not individually identifiable and therefore no informed consent was required for their use for *in vitro* diagnostic evaluation.

### Role of the funding sources

This work was supported by the USAID grant GHA-G-00-07-00007 and ZikaPLAN (European Union's Horizon 2020 Research and Innovation Programme under Grant Agreement No. 734584). The funders have no role in the writing of the manuscript nor the decision to publish the results. The corresponding author, RWP, have accessed and verified the data, and RWP and DB were responsible for the decision to submit the manuscript.

## Results

All but one test passed the initial Sudden Death panel. The test that failed was allowed to be re-tested after the manufacturer adjusted the test cut-off. When this did not improve the test performance, the test was disqualified from the full evaluation. Full evaluation was performed for three RDTs.

### The Chembio DPP® Zika IgM/IgG test and the DPP ZCD IgM/IgG system

A summary of the performance of the Chembio DPP® ZIKV IgM/IgG test is shown in Table 3. The sensitivity and specificity of the test for anti-ZIKV IgM antibodies was 86.4% and 87.2%, respectively. A summary of the performance of the Chembio DPP® ZCD IgM test is shown in Table 4. The sensitivity/specificity for anti-ZIKV IgM was 79.0%/97.1%, for anti-DENV IgM was 90.0%/89.2%, and for anti-CHIKV IgM was 90.6%/97.2%. The DPP IgM test initially showed reactivity against 47% specificity against 7 malaria positive specimens, 92.3% against 13 rheumatoid positive specimens and 80% against 10 healthy controls. The company was able to overcome these non-specific binding by internal adjustment of the assay so that these problems were no longer observed with both DPP ZIKV and ZCD IgM tests.

**Table 3:** Summary of the Performance of the Chembio DPP® Zika IgM test.

Samples	ZIKV IgM	
	Sensitivity	Specificity
Anti-ZIKV IgM	**86.4 % (72.6–94.8) [**38/44]	
**Specificity**		
Anti-DENV IgM		89.9 % (82.2-95) [89/99]
Anti-YFV IgM		73.3 % (44.9-92.2) [11/15]
Anti-CHIKV IgM		71.4% (29-96.3) [5/7]
**Specificity vs Arboviruses**		**86.7% (78.5-91.6) [105/121]**
-vs Anti-ZIKV IgG		80% (44.4-97.5)[8/10)]
-vs Healthy controls		100% (94.4-100)[10/10]
**Overall Specificity**		**87.2% (76.3-88.2) [123/141]**

ZIKV: zika virus; DENV: dengue virus; CHIKV: chikungunya virus; YF: Yellow Fever virus.

() = 95% confidence intervals.

[] = number of specimens tested positive/total number of specimens tested for sensitivity; number of specimens tested negative/total number of specimens tested for specificity.

**Table 4:** Summary of the performance of the Chembio DPP® ZCD IgM test.

	ZIKV IgM	DENV IgM	CHIKV IgM
**Sensitivity**	**79.0% (72.1–84.8)** [135/171]	**90.0% (84.5–94.1)** [153/170]	**90.6% (85.8–94.1)** [192/212]
**Specificity**			
**-** vs ZIKV	NA	90.0% (73.5**–**97.9) [27/30]	98.8% (93.3**–**100) [80/81]
**-** vs DENV	94.3% (84.3**–**98.8) [50/53]	NA	96.3% (81**–**100) [26/27]
**-** vs CHIKV	98.4% (91.3**–**100) [61/62]	78.6% (49.2**–**95.3) [11/14]	NA
**-** vs YFV	95.4% (77.1**–**100) [21/22]	72.7% (49.8**–**89.3) [16/22]	90.9% (70.8**–**98.9) [20/22]
**Specificity vs Arboviruses**	**96.3% (90.7–98.4)** [132/137]	**81.8% (70.4–90.2)** [54/66]	**96.9% (92.3–99.1)** [126/130]
**-** vs ZIKV IgG	100% (69.1**–**100) [10/10]	NA	NA
**-** vs healthy controls	100% (94.8**–**100) [69/69]	100% (92.1**–**100) [45/45]	98.0% (89.3**–**100) [49/50]
**Overall specificity**	**97.1% (93.8–98.9)**	**89.2% (81.9–94.3)**	**97.2% (93.6–100)**

NA: not applicable.

ZIKV: zika virus; DENV: dengue virus; CHIKV: chikungunya virus; YF: Yellow Fever virus.

() = 95% confidence intervals.

[] = number of specimens tested positive/total number of specimens tested for sensitivity; number of specimens tested negative/total number of specimens tested for specificity.

### The SD Biosensor STANDARD Q Arbo I Panel (Z/D/C/Y) IgM Tests

A summary of the performance of the SD Biosensor STANDARD Q Arbo I Panel (Z/D/C/Y) IgM Tests across all sites is shown in Table 5**.** The sensitivity/specificity for anti-ZIKV IgM test was 96.8%/90.8%, for anti-DENV IgM test was 71.8%/83.5%, for the DENV NS1 glycoprotein was 90.0%/90.2%, for anti-YFV IgM was 84.6%/92.4%, and for anti-CHIKV IgM was 86.3%/97.5%.

**Table 5:** Summary of the Performance of the SD Biosensor STANDARD Q Arbo Panel Test.

	ZIKV IgM	DENV IgM	DENV NS1	YFV IgM	CHIKV IgM
**Sensitivity**	**96.8%** **(88.8–99.0)** [60/62]	**71.8%** **(64.2–78.5)** [117/16])	90%* [9/10]	**84.6%** **(80.2–91.1)** [33/39]	**86.3%** **(80.2–91.1)** [145/168]
**Specificity**					
**-** vs ZIKV	NA	62.9% [39/62]	100% [16/16]	80.0% [40/50]	98.4% [61/62]
**-** vs DENV	86.7% [52/60]	NA	NA	92.3% [36/39]	100% [60/60]
**-** vs YFV	68.8% [11/16]	68.8% [11/16]	61.5% [8/13]	NA	100% [7/7]
**-** vs CHIKV	98.7% [77/78]	98.7% [78/79]	100% [9/9]	100% [73/73]	NA
**Specificity vs Arboviruses**	**90.9%** [140/154]	**81.5%** [128/157]	**89.1%** [41/46]**	**92.0%** [149/162]	**99.3%** [135/136]
**-**vs IgG	80% [8/10]	100% [9/9]	**–**	**–**	77% [10/13]
**-**vs healthy controls	100% [10/10]	100% [10/10]	100% [10/10]	100% [10/10]	100% [10/10]
**Overall specificity**	**90.8% (85.5–94.6)**	**83.5%** **(73.8–86.5)**	90.2%**	**92.4%** **(87.4–95.9)**	**97.5%** **(94.9–99.8)**

*n=10 dengue PCR+ (*n* = 10); **also tested against malaria 100% (0/5) and West Nile PCR+ 100% (0/8).

ZIKV: zika virus; DENV: dengue virus; CHIKV: chikungunya virus; YF: Yellow Fever virus.

() = 95% confidence intervals.

[] = number of specimens tested positive/total number of specimens tested for sensitivity; number of specimens tested negative/total number of specimens tested for specificity.

In summary, the sensitivity of the three anti-ZIKV IgM antibody RDTs ranged from 79.0 to 96.8% while specificity ranged from 90.8 to 97.1% with tests showing the highest sensitivity having the lowest specificity and vice versa. The sensitivity and specificity of the anti-DENV IgM tests ranged from 71.8% to 90% and 80.7% to 89.2%, respectively. Anti-DENV IgM tests has been recommended to be used in combination with DENV NS1 antigen tests to increase the sensitivity of detection of acute dengue infection.^34^^,^^35^ The DENV NS1 test in this evaluation showed a sensitivity of 90.0% and a specificity of 90.2%. Both the anti-CHIKV and anti-YFV IgM antibody tests showed sensitivities and specificities above the TPP recommendations of ≥85%.

## Discussion

Rapid and accurate diagnostics are essential during disease outbreaks to identify cases and guide patient management, map the extent of the outbreak and monitor the effectiveness of interventions.^2^ However, sustaining industry interest to develop tests truly suitable for such purposes has been difficult.^1^ For diseases of epidemic potential, diagnostic companies are often hesitant to invest in research and test development due to inconsistent demand and uncertain market size. Since outbreaks of infectious diseases are occurring more frequently and with increased severity, setting up innovative mechanisms to incentivise test development and biobanking networks to ensure access to well-characterised specimens to facilitate test development and evaluation reduces the risks companies face investing in diagnostics of epidemic potential and enable countries to have improved tools for clinical management and surveillance providing early alerts of disease outbreaks.

On the basis of the evaluation described here along with financial and other considerations, the Chembio ZIKV IgM/IgG RDT, the Chembio ZCD IgM/IgG RDT and the SD BioSensor Standard Q Arbo I Panel were recommended by the UNICEF Procurement Reference Group for purchase under the UNICEF-USAID APC mechanism. This evaluation was expedited through a network of biobanking sites with well characterised samples already established through an EU-funded ZikaPLAN project. The variety of biobanking sites ensures that specimens used for this evaluation comprised of specimens collected from primary and secondary arbovirus infections through outbreak investigations, from sentinel surveillance sites and from research studies in countries where these RDTs would be used. This evaluation provided a template for setting up biobanking and evaluation mechanisms for epidemic preparedness which was rapidly adapted for the COVD-19 pandemic by the Africa Centres for Disease Control and Prevention.^23^

The anti-ZIKV IgM sensitivity of 79% observed with the Chembio DPP® ZCD IgM test was lower than the 86.4% observed for the ChemBio DPP Zika IgM/IgG test. This could partly be the cut-off for a multiplex test has to be balanced with the other tests in the system. The higher specificity for the ChemBio ZCD IgM RDT of 97% versus that of 86% for the singlex test came at the expense of a lower sensitivity of 79% compared to 86% for the singlex IgM tests. These thresholds can be further adjusted with more field experience of using these tests.

The SD BioSensor anti-DENV IgM showed suboptimal sensitivity of 71.8% and specificity of 80.7% but its DENV NS1 antigen (Ag) detection test showed excellent sensitivity and specificity for the detection of acute DENV infections. Studies have shown that combined use of an anti-DENV IgM plus NS1 Ag test can result in highly accurate detection of acute DENV infections with longer detection window than either test used alone.^34^^,^^35^

For this tender, UNICEF adopted the WHO TPP for Zika diagnostics and set mandatory requirements of 85% for sensitivity and specificity of ZIKV singleplex and multiplex tests compared to a laboratory reference standard. Hence, the Chembio DPP® Zika IgM/IgG, Chembio DPP® ZCD IgM/IgG test and SD Biosensor STANDARD Q Arbo I (Z/D/C/Y) Panel test should all be considered acceptable for multiple use cases, such as an aide to diagnosis and surveillance to provide outbreak alerts. As these RDTs are intended only as an aid to diagnosis and for surveillance, positive results should be confirmed with more specific laboratory-based assays for a definitive clinical diagnosis and as part of outbreak investigations. The specificity of these tests is expected to be much better in real life settings since not all cross-reactive conditions used in the challenge panel would be expected in any one geographic location. UNICEF has started to pilot studies to determine the feasibility of using these RDTs for ZIKV surveillance at both arboviral sentinel surveillance sites and at antenatal clinics. It is anticipated that the results of these studies will inform whether these tests are satisfactory to aid clinical management and for surveillance.

The current evaluation has limitations in terms of obtaining a sufficiently large sample size for some of the sensitivity and specificity panels, especially for anti-YFV IgM positive specimens. It is anticipated that the biobanking network will continue to expand to improve both in terms of specimens available for evaluation as well as better geographical representation. On the other hand, the advantage of the evaluation process described for this APC mechanism is that it leverages the combined power of a network of quality sites to contribute well-characterised archived specimens towards an evaluation without costly transport of specimens across borders or potential compromise of sample quality in transport. This mechanism allows the rapid evaluation of new tests that are developed in response to an outbreak of emerging, re-emerging or novel pathogens of epidemic potential.  It is not meant to replace more in-depth evaluations of the clinical performance or clinical utility of tests that would still be required when time allows.

According to the WHO, The global incidence of dengue has grown dramatically in recent decades with about half of the world's population now at risk.^36^ The disease is endemic in more than 100 countries. DENV and ZIKV co-circulation will continue to pose differential diagnostic challenges. Initiatives to continually improve the performance of rapid serology tests that can be used in the field to accurately distinguish between ZIKV, DENV, CHIKV, and YFV and support surveillance to inform control strategies should be urgent priority. In many LMICs access to conventional highly specific techniques to confirm the diagnosis is limited. Thus, RDTs that are adequately sensitive and specific but widely accessible would be useful for clinical medicine at the point-of-care within communities and for surveillance, providing early alerts of outbreaks.

This UNICEF-USAID APC mechanism to fund the development and evaluation of more accurate and accessible ZIKV tests after ZIKV is no longer a PHEIC is an important and much needed initiative. The results reported here show that the UNICEF APC mechanism did achieve its goal of ensuring that countries have access to affordable and accurate RDTs to respond to future outbreaks of ZIKV and other arboviral infections. Field studies to determine the feasibility of using these RDTs for arbovirus case detection and surveillance, including prenatal care settings, are ongoing. This mechanism can serve as a template for epidemic preparedness for other diseases of epidemic potential.

## Contributors

RWP and DB designed the evaluation in collaboration with all the other authors who conducted the evaluations and analysed their site-specific data. RWP, CTD and DB collated all the site-specific data and performed the analysis for review by all authors. All authors have access to the data and the results of the analyses. RP and DB took the decision to submit the manuscript for publication. All authors contributed to the writing and finalisation of the manuscript.

## Declaration of interests

We declare that we have no conflicts of interest.

## Data sharing statement

Requests for data sharing should be submitted to the corresponding author.
